# Neurotoxicological Evaluation of Intrathecal Citrate Excipients: Calcium Homeostasis Disruption and Safety Implications in CNS Drug Delivery

**DOI:** 10.3390/pharmaceutics17091112

**Published:** 2025-08-27

**Authors:** Jue Wang, Yuan Zhang, Qing He, Ying Du, Xia Zhang, Xinru Tan, Xinting Zhou, Susu Tang, Baoming Ning, Rui Yang, Xia Zhao, Dejiang Tan, Huimin Sun, Jiasheng Tu

**Affiliations:** 1 Center for Research Development and Evaluation of Pharmaceutical Excipients and Generic Drugs, Department of Pharmaceutics, School of Pharmacy, China Pharmaceutical University, Nanjing 210009, China; wangjue2020@nifdc.org.cn (J.W.);; 2 National Institutes for Food and Drug Control, Beijing 100050, Chinaningbm@nifdc.org.cn (B.N.); yangr@nifdc.org.cn (R.Y.);

**Keywords:** intrathecal administration, sodium citrate, neurotoxicity, calcium homeostasis, excipient safety

## Abstract

**Background/Objectives:** Intrathecal drug delivery is essential for treating CNS disorders, but the safety of commonly used excipients such as citric acid/sodium citrate (SC) remains unclear. This study aims to systematically evaluate the potential neuropharmacological effects of repeated intrathecal SC administration. **Methods:** Multimodal approaches were applied across murine and lagomorph models. Doses ranged from 1.833–14.664 μg/g in mice and 0.104–3.290 mg/rabbit. Behavioral, neurophysiological, and fiber photometry analyses were conducted to assess sensorimotor function, cortical activity, and calcium dynamics. **Results:** SC induced dose-dependent sensorimotor deficits, including hypolocomotion (45.7% reduced distance, *p* < 0.001) and impaired coordination (latency reduction 48.3–64.1%, *p* < 0.001). Mortality increased with dosage and repeated exposure. Neurophysiological data revealed biphasic cortical modulation: acute c-Fos suppression followed by delayed hyperactivity. Fiber photometry confirmed calcium chelation-mediated attenuation and subsequent potentiation of Ca^2+^ signals. Rabbits exhibited similar neurological symptoms, correlating with transient CSF calcium/magnesium depletion, though no structural neural damage was observed. **Conclusions:** These results provide the first comprehensive evidence that SC buffers can significantly disrupt neuronal calcium homeostasis and induce functional impairments upon intrathecal delivery. The findings emphasize the need for reassessing excipient safety in CNS-targeted formulations.

## 1. Introduction

Intrathecal administration, a method of delivering drugs directly into the subarachnoid space of the spinal cord [1], is primarily employed in scenarios requiring rapid drug delivery to the central nervous system (CNS) or the circumvention of the blood–brain barrier [2]. Consequently, it is frequently utilized in the management of CNS-related disorders, including pain, spasticity, cancer chemotherapy, and infections [3,4,5,6]. However, an intrathecal injection is a highly specialized procedure that demands stringent aseptic conditions [7] and precise dosage control [8] to avoid severe adverse effects, such as infections, neural damage, or respiratory depression [9,10,11]. In clinical practice, due to the complexity of the procedure, adverse reactions are often attributed to mechanical manipulation or injection techniques [12], while issues related to the formulation itself, particularly the excipients, are frequently overlooked. Notably, many drugs used for intrathecal administration fall under “off-label use” [13,14], meaning they are not explicitly approved for this route but are widely utilized in clinical settings [15]. In recent years, with the increasing demand for CNS disease treatments, a growing number of intrathecal drugs have been under development or have been marketed [16,17,18], such as Nusinersen (Spinraza^®^) for spinal muscular atrophy (SMA), which acts directly on spinal motor neurons via intrathecal delivery [19]. Additionally, novel intrathecal formulations for chronic pain management are also being developed [20].

Despite these advancements, there remains insufficient attention to whether pharmaceutical excipients in these formulations are suitable for intrathecal administration and whether incompatibility issues may arise due to the unique physiological environment of the injection site. If these concerns are not adequately addressed, they could pose significant risks [21], particularly for vulnerable patient populations such as children, where the potential hazards may be amplified [22,23,24,25].

Citric acid and sodium citrate are commonly used pharmaceutical excipients in injectable formulations, primarily serving as pH adjusters, metal ion chelators, and stabilizers to enhance drug stability and solubility and reduce irritability [26,27]. However, the safety of their application in intrathecal administration, a route that directly targets the CNS, has not been thoroughly evaluated [28]. In neurological research, rodent models (particularly mice) are well-established as the most prevalent experimental systems, while rabbits serve as representative mid-sized animals for supplementary validation. Therefore, in this study, we administered intrathecal injections of citric acid and sodium citrate solutions in mice and rabbits, reporting dose-dependent behavioral changes in mice and reactive responses in rabbits. Furthermore, we assessed calcium ion concentrations in specific brain regions of mice post-injection, providing preliminary insights into the safety and mechanisms of the intrathecal citric acid and sodium citrate administration.

## 2. Methods and Materials

### 2.1. Animals

ICR mice (6–8 weeks, half male and half female) were acquired from the Medical Center of Yangzhou University (Yangzhou, China) and housed in a stable environment with 23 ± 2 °C temperature, 55 ± 5% humidity, and a 12 h/12 h light/dark cycle (lights on at 7:00 am) for 7 days before the experiment. Mice ate food and drank water freely. New Zealand rabbits (conventional grade; 2–4 months old; 2.1–2.6 kg body weight) of both sexes (50% male, 50% female) were used in this study. The animals, sourced from Kangda Aibo Biotechnology Co., Ltd. (Qingdao, China)—an accredited laboratory animal provider (Quality Certification No. 370823240100197621)—were non-transgenic wild-types and serologically confirmed as pathogen-free. All rabbits were individually housed in stainless-steel cages supplemented with environmental enrichment (e.g., toys) under standardized conditions (SOP-AC-087.02, Jiangsu Dingtai Pharmaceutical Research Co., Ltd. Nanjing, China). Animals received commercial diet (Ke’ao Xieli Feed Co., Ltd., Beijing, China) ad libitum. Following a 7-day acclimation period, experimental procedures were initiated. The mice experimental procedures were approved by the Animal Ethics Committee of China Pharmaceutical University (Ethics Code 2024-12-040), and the rabbit study proposal was approved by the Ethics Committee of National Institutes for Food and Drug Control with the permit number AP-24121-450N.

### 2.2. Intrathecal Injection

Each mouse was anesthetized with 5% isoflurane (RWD Life Science, Shenzhen, China). After the mice were completely anesthetized (the righting reflex was lost and the tail clip did not produce violent movement), the hair on the back of the mice was removed with an electric hair shaver, and the skin on the back of the mice was gently cut with scissors. The needle was inserted vertically into the middle of the most prominent place of the spinal column, and the angle was reduced to approximately 30° after touching the bone, then the needle advanced into the vertebral space at L5–L6. Obvious tail flutter indicates the needle has been successfully inserted into the vertebral space. Drug solutions (1.833, 3.666, 7.332, or 14.664 μg/g citrate equivalents) or saline were intrathecally administered at 520 nL/g via syringe pump over 30 s. The 3.666 μg/g dose represents the human equivalent of 28.2 mg via body surface area conversion. After the injection, the wounds of mice were sutured with absorbable sutures and disinfected with iodophor. Three days later, the mice received a second intrathecal injection.

One day prior to the experiment, the dorsal lumbar region of each New Zealand White rabbit was shaved using electric clippers. On the experimental day, anesthesia was induced with 5% isoflurane and maintained with 3% isoflurane. Under anesthesia, an intrathecal injection was administered between the L5–L6 vertebrae, where test compounds (0.104, 0.292, 1.175, or 3.290 mg/rabbit) or saline (0.1 mL/rabbit) were slowly infused over 5 min before gradual needle withdrawal. For pre-dosing cerebrospinal fluid (CSF) collection, approximately 0.5 mL CSF was obtained via cisterna magna puncture using the same anesthesia protocol. Post-dosing CSF collection employed anesthesia through intramuscular injection of ketamine hydrochloride (30 mg/kg) and xylazine hydrochloride (3 mg/kg). Following final CSF collection, euthanasia was performed via femoral artery exsanguination. Spinal cord tissue surrounding the injection site and corresponding dorsal root ganglia were harvested, fixed in 10% neutral buffered formalin, then processed through dehydration, paraffin embedding, sectioning, hematoxylin and eosin (H&E) staining, and coverslipping for histological examination. All formulations maintained physiological osmolarity (293–296 mOsm/kg) and pH (7.3 ± 0.2) through controlled saline/water ratios and citric acid adjustment.

### 2.3. Behavioral Tests

All the mice were habituated to the experimental room 30 min before the test. After each behavioral test, 75% ethanol was used to clean the experimental apparatus. All the tests were performed by a technician blinded to the treatment group, and the data were recorded using the video tracking system (v0.9.11, Jiliang, China). Additionally, we assessed physiological responses and mortality in mice at three time points: immediately post-injection, 1 h, and 24 h after intrathecal administration.

#### 2.3.1. Open Field Test (OFT)

The open field activity monitoring system comprehensively assesses locomotor and behavioral activity levels of mice. It is primarily used to assess spontaneous activity levels, anxiety behavior, exploratory behavior, and motor ability of experimental animals [29,30]. The experimental device is composed of a 50 cm × 50 cm × 40 cm square box without cover and a video acquisition system. Each mouse was placed into the center of the open rectangle field and allowed to explore the apparatus for 5 min freely. The digital video tracking system recorded the average speed and total distance in the OFT.

#### 2.3.2. Grid Test (GT)

Grid testing can assess an animal’s motor coordination, balance, and movement functions of both front and hind paws, serving as a crucial basis for studying neurological diseases, drug efficacy, and neural injury repair [31,32]. The experimental device is composed of a 50 cm × 50 cm × 40 cm square box without cover, a 60 cm × 60 cm hard metal grid plate, and a video acquisition system. In a quiet environment, the mice were placed upside down on a grid plate for 3 min. The latency of drop for the mice from the grid board to the box for the first time was recorded. For the mice staying on the grid board until the end of the time, the latency was recorded as 3 min.

#### 2.3.3. Hot Place Test (HPT)

The Hot Plate Test (HPT) is a method used to evaluate animals’ pain responses to heat stimuli, assessing their pain thresholds and the efficacy of analgesic drugs [33]. In this study, the mice were placed on a plate heated to 55 °C (hot plate) for 30 s. The latency of paw lifting and the number of thermal reactions (lift, lick, jump) in 30 s were recorded.

### 2.4. Immunofluorescence

The mouse brains were taken out, respectively, 10 min or 60 min after the second intrathecal injection. Mice were perfused transcardially with phosphate-buffered saline (PBS) and 4% paraformaldehyde. Then brains were removed, fixed in 4% paraformaldehyde overnight, and dehydrated in 30% sucrose for 2 days. For frozen slices, 25 μm or 30 μm coronal sections were sliced on a freezing microtome (CM1950, Leica, Germany). After incubating with 10% donkey serum ((SL050, Solarbio Life Science, Beijing, China), the brain slices were incubated in rabbit anti-c-Fos (1:400, 226008, Synaptic Systems, Germany) in a refrigerator at 4 °C overnight. After the incubation of primary antibody, the brain slices were washed in PBS 3 times for 5 min each time. Donkey anti-rabbit Alexa Fluor 488 (1:500, 33106ES60, Yeasen Biotechnology, Shanghai, China) was selected as the second antibody and incubated in the dark at room temperature for 2 h. After the second antibody incubation, the brain slices were washed in PBS 3 times, 5 min each time. Then the DAPI solution (C0065, Solarbio Life Science, China) was incubated to stain nuclei in the dark at room temperature for 5 min. All immunofluorescent images were obtained by fluorescence microscope (DM2000, Lecia, Wetzlar, Germany). Then, the cell staining was counted using Leica Application Suite X software by an individual experimenter blind to the experiment.

### 2.5. Stereotaxic Injection

Each mouse was anesthetized with 5% isoflurane and kept anesthetized with 2.5% isoflurane. Then the mouse was positioned in a stereotaxic frame (Harvard Apparatus, Holliston, MA, America). The calcium indicator adeno-associated virus vector (AAV-hSyn-GCamp6s, BrainVTA, Wuhan, China) carrying gene coding at a volume of 200 nL was microinfused into the target regions at a flow rate of 50 nL/min with a microinjection pump (Harvard Apparatus, Holliston, MA, USA). The microliter syringe (Gaoge, Shanghai, China) was left at the injection site for 5 min to ensure spreading virus totally after the microinjection. The coordinates of brain region were as follows: AP 1.78 mm, ML ±0.30 mm, DV −2.35 mm. AP, ML, and DV, successively represented the anterior–posterior, medial–lateral, and dorsal–ventral positions relative to the bregma.

### 2.6. Fiber Photometry

Fiber photometry is a novel optogenetic method for recording neural activity in vivo. It enables in vivo neural activity recording by transforming cellular calcium concentration fluctuations into detectable fluorescent signals. These signals are then captured by the fiber photometry system, allowing for real-time observation of neural activity [34]. In our study, 200 nL AAV-hSyn-GCaMP6s was bilaterally injected into prelimbic cortex (PrL). Two weeks later, ceramic ferrule with optical fiber (200 μm in diameter, NA 0.24, Inper, Suzhou, China) was implanted above the PrL and was fastened using denture base materials (Sncdental, Shanghai, China). After 3 days of implantation, calcium signals were recorded by fibro photometry system (Inper Dataprocess v0.7.2, Suzhou, China) 0–10 min and 60–70 min after the second intrathecal injection. Set the excitation light to steady on mode; that is, set the sampling rate and exposure to the maximum, adjust the 405 nm light intensity to 10 μW, 470 nm light intensity to 30 μW, then set the sampling rate to 60 Hz, and set the exposure time to 10 ms. The original data were corrected by Inper Data Process software v0.7.2. We analyzed area under curve (AUC) and the ΔF/F_0_: ΔF/F_0_ (%) = [(F − F_0_)/F_0_ × 100]. The heatmaps and average plots were performed by MATLAB (R2019b, MathWorks, Natick, MA, USA) and Adobe Illustrator (CC2018, Adobe, San Jose, CA, USA).

### 2.7. Statistical Analysis

GraphPad Prism 9 software was used for statistical analysis. Student’s *t*-tests were used for two groups: one-way ANOVA and two-way ANOVA with post hoc comparison for multiple comparisons. All data were expressed as mean ± standard deviation (SD). Significance levels were indicated as *^ns^ p* > 0.05, * p < 0.05, ** p < 0.01, and *** p < 0.001.

## 3. Results

### 3.1. Intrathecal Injection of Citric Acid/Sodium Citrate (SC) Causes Hind Limb Injury in Mice

**Figure 1:**
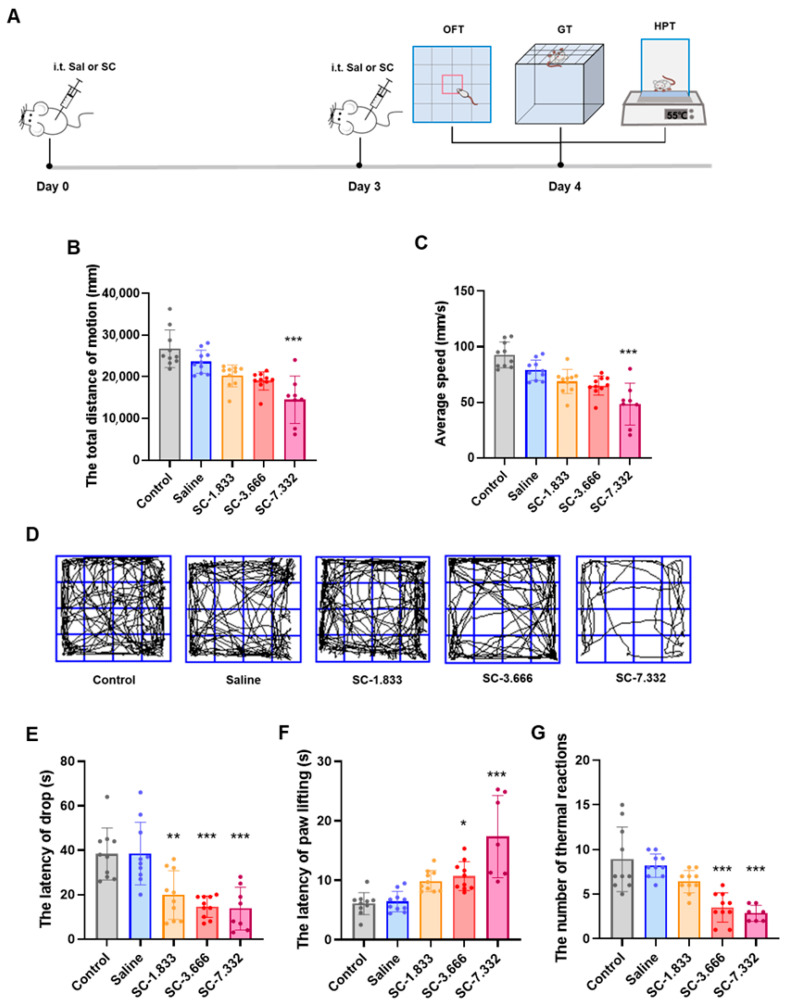
(**A**) Experimental flowchart; (**B**) total distance traveled by mice in each group in the Open Field Test; (**C**) average speed of mice in each group in the Open Field Test; and (**D**) representative trajectory plots of movement by mice in each group in the Open Field Test. (**E**) The latency to fall in the Grid Test for mice in each group. (**F**) The paw lifting latency of mice in each group in the Hot Plate Test; (**G**) the number of thermal responses within 30 s in the Hot Plate Test for mice in each group. n = 8–10; *^ns^ p* > 0.05, * *p* < 0.05, ** *p* < 0.01, and *** *p* < 0.001 compared to the saline group.

To better simulate clinical drug administration scenarios, we adopted a repeated dosing regimen, administering a second dose to the mice three days after the initial injection. Following two intrathecal administrations of citric acid/sodium citrate (SC) at graded concentrations (1.833, 3.666, 7.332 μg/g) or equivalent-volume saline (n = 20/group) over 48 h, comprehensive neurobehavioral assessments were conducted using standardized paradigms: the Open Field Test (OFT; 5 min observation in 50 × 50 cm arena), Grip Traction Test (GT), and Hot Plate Test (HPT; 55 ± 0.5 °C). The quantitative analysis revealed dose-dependent neurofunctional impairments in both sensorimotor domains (Figure 1B,C,E–G).

In Open Field Test evaluations, the 7.332 μg/g SC group showed significantly reduced locomotor activity, with a 45.7% decrease in the total movement distance and a 47.8% reduction in the average velocity compared to normal controls (*p* < 0.001), as evidenced by the representative movement trajectories shown in Figure 1D. The Grid Test revealed progressive motor coordination deficits across SC doses, with the fall latency significantly shortened by 48.3% (1.833 μg/g, *p* < 0.001), 61.9% (3.666 μg/g, *p* < 0.001), and 64.1% (7.332 μg/g, *p* < 0.001) relative to controls (Figure 1B,C). The intrathecal administration of 3.666 μg/g and 7.332 μg/g of SC significantly increased the paw withdrawal latency and decreased thermal nociceptive responses in the Hot Plate Test (Figure 1F,G), demonstrating the dose-dependent impairment of both sensory perception and motor function in mouse hind limbs. These findings collectively indicate that the intrathecal SC injection induces concentration-dependent deficits in sensorimotor performance.

### 3.2. Intrathecal Injection Induces Dose-Dependent Behavioral Responses in Mice

We evaluated the mice at three time points: during each administration (Imm.), 1 h post-administration, and 24 h post-administration. Observations included the tail twisting, convulsions, hindlimb rigidity, hindlimb weakness, locomotor abnormality and recovery time, with both the initial number of mice and the survival count also documented, as outlined in Table 1.

The experiment revealed a pronounced dose-dependent toxicity. Following the first administration, the 7.332 μg/g dose group exhibited a 30% mortality rate (6/20), while the 14.664 μg/g group experienced 100% mortality (20/20). After the second administration, mortality was observed even at the lower dose of 3.666 μg/g (10%, 2/20), and the 7.332 μg/g group suffered an additional six deaths, resulting in a cumulative mortality rate of 60% (12/20). The time series analysis demonstrated a strong linear correlation between mortality and the dose after the first administration (r = 0.98). Notably, the toxicity was significantly exacerbated upon the repeated administration, particularly at doses exceeding 7.332 μg/g.

### 3.3. Neurophysiological Response to Intrathecal SC Administration: c-Fos Immunohistochemical Analysis

To investigate the temporal dynamics of the neuronal activation following the intrathecal SC administration, we performed an immunohistochemical analysis of the c-Fos expression, a well-established marker of neuronal activity, in key brain regions associated with motor control and sensory processing. Our time-course evaluation revealed a biphasic modulation of neuronal activation patterns.

**Figure 2:**
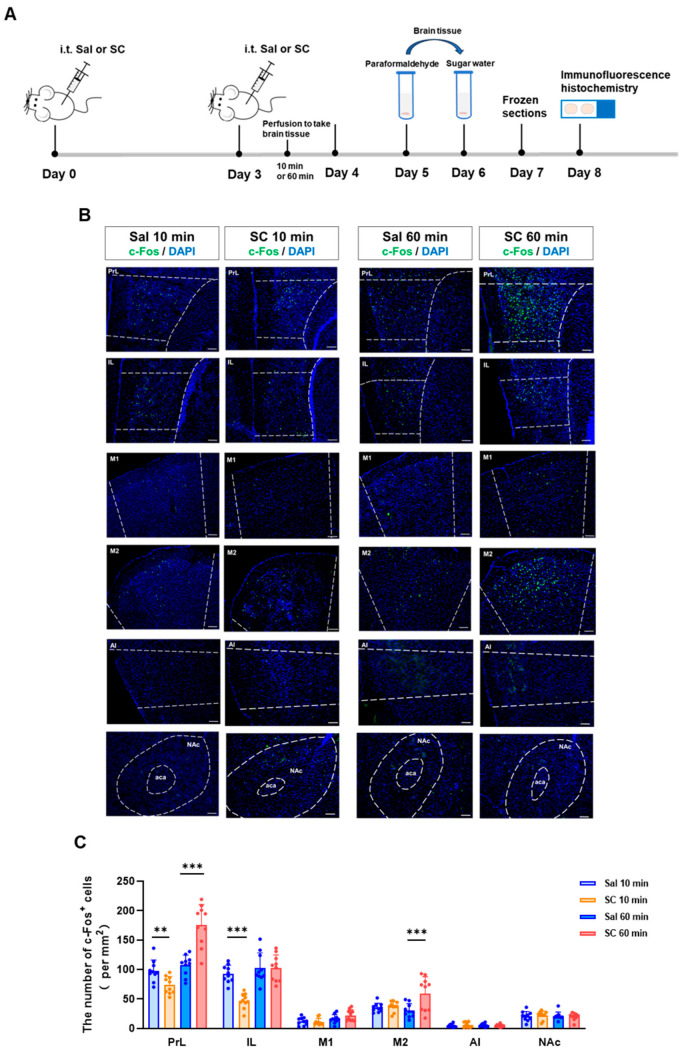
Effects of intrathecal administration of citric acid/sodium citrate on neuronal activity in motor- and sensory-related brain regions in mice. (**A**) Experimental flowchart; (**B**) representative fluorescence images of c-Fos expression in the PrL, IL, M1, M2, AI, and NAc brain regions of mice in each group (green indicates c-Fos fluorescence signal, and blue indicates nuclear DAPI staining), scale bar = 100 μm; and (**C**) statistical graphs of c-Fos expression in each brain region. n = 10, ** *p* < 0.01, and *** *p* < 0.001.

At the acute phase (10 min post-injection), we observed a significant suppression of neuronal activity, as evidenced by reduced c-Fos+ cell counts in both the prelimbic cortex (PrL) and the infralimbic cortex (IL) (*p* < 0.05 vs. saline controls). This initial inhibitory phase was followed by a subsequent excitatory response, with the marked elevation of the c-Fos expression in both the PrL and secondary motor cortex (M2) at 60 min post-administration (*p* < 0.01) (Figure 2B,C).

These temporal patterns of neuronal activation demonstrate that the intrathecal SC administration induces a biphasic neuromodulatory effect, characterized by an initial inhibitory phase followed by delayed neuronal excitation. The differential activation patterns across distinct cortical regions (PrL, IL, and M2) suggest a region-specific sensitivity to the SC modulation, potentially reflecting the compound’s complex pharmacological actions on neural circuits involved in sensorimotor integration.

### 3.4. Fiber Photometry Reveals Biphasic Modulation of PrL Neuronal Activity Following Intrathecal SC Administration

To investigate whether the intrathecal SC administration affects neuronal activity through calcium ion chelation mechanisms, we employed fiber photometry to monitor real-time calcium dynamics in the prelimbic cortex (PrL). It is known that the vicinal diol group in SC’s structure exhibits a strong metal ion chelating capability [35], particularly demonstrating a nanomolar-level affinity (Kd) for Ca^2+^. We hypothesized that SC may competitively bind intracellular Ca^2+^, thereby disrupting calcium-dependent signaling pathways and producing temporally specific neuromodulatory effects [36,37].

Adult C57BL/6 mice were stereotaxically injected with AAV-hSyn-GCaMP6s into the PrL to enable genetically encoded Ca^2+^ indicator expression. After a 2-week recovery period to allow for sufficient viral expression, an optical fiber was implanted above the PrL. Behavioral experiments commenced 3 days post-implantation to ensure a stable fiber attachment and minimize surgical stress effects.

**Figure 3:**
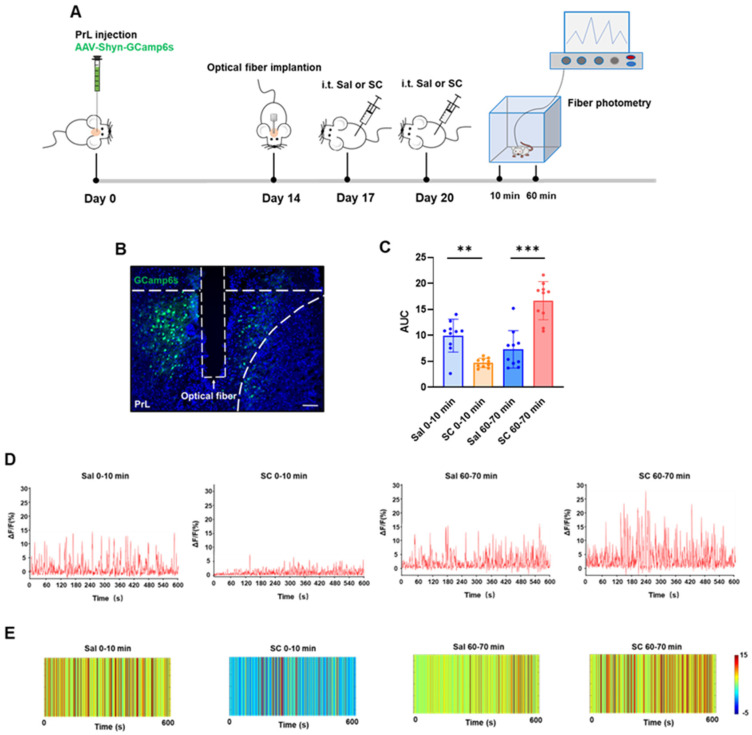
Effects of intrathecal injection of citric acid/sodium citrate on calcium ion signaling in the PrL brain region of mice. (**A**) Experimental flowchart; (**B**) schematic diagram of viral expression (green) and fiber optic implantation, scale bar = 100 μm; (**C**) statistical values of the area under the calcium signal curve in the PrL brain region of mice in each group; (**D**) representative linear plots of calcium signals in the PrL brain region of mice in each group; and (**E**) representative heatmaps of calcium signals in the PrL brain region of mice in each group.

The neuronal activity was recorded during two critical time windows (0–10 min and 60–70 min post-injection) following the second intrathecal SC administration to evaluate both acute and delayed responses. As shown in Figure 3, compared with the saline control group, the intrathecal administration of citrate/citrate sodium elicited a biphasic modulation of calcium dynamics in the PrL region: during the acute phase (0–10 min post-injection), we observed a significant attenuation of calcium signals accompanied by a marked reduction in the area under the curve (AUC) (*p* < 0.01), whereas the delayed phase (60–70 min post-injection) exhibited a pronounced potentiation of calcium signals with a significantly increased AUC (*p* < 0.001). These data demonstrate that the intrathecal citrate administration induces temporally distinct effects on the PrL calcium homeostasis, characterized by an initial 10 min period of a Ca^2+^ level reduction followed by a significant Ca^2+^ elevation after 60 min.

This temporal pattern suggests multifaceted SC actions on cortical circuits, potentially mediated through the following: (1) immediate receptor-mediated inhibition, (2) secondary network-level disinhibition [38], and (3) delayed transcriptional/neuromodulatory mechanisms [39]. The PrL’s differential responsiveness likely stems from its unique integrative role within the cortico-limbic circuitry, processing convergent sensory, motor, and affective information. These electrophysiological findings not only corroborate our c-Fos data (Figure 2) but also provide direct functional evidence for SC’s phase-specific neuromodulatory properties, revealing complex spatiotemporal dynamics in cortical information processing following SC exposure.

### 3.5. Post-Intrathecal Administration Clinical Phenotypes in Rabbit Models

In order to validate the dose-dependent neuromodulatory effects observed in murine models and assess their relevance in a species with a closer neuroanatomical and pharmacokinetic resemblance to humans, we further conducted parallel experiments in rabbit models.

**Figure 4:**
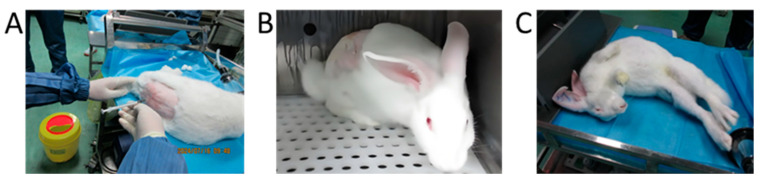
Behavioral manifestations of intrathecal sodium citrate injection in rabbits. (**A**) Photographs of intrathecal injection in rabbits; (**B**) 0.292 mg/rabbit intrathecal injection of sodium citrate showed no abnormal reaction; (**C**) 3.290 mg/rabbit intrathecal injection of sodium citrate immediately after abnormal reaction (stiffness of hind legs, etc.).

In this investigation, four intrathecal sodium citrate dose groups were established in rabbits: 0.104, 0.292, 1.175, and 3.290 mg/rabbit (n = 6 per group, equal gender distribution). Rabbits in the lower-dose cohorts (0.104 and 0.292 mg/rabbit) demonstrated no observable abnormalities (Figure 4B). However, dose-dependent neurobehavioral alterations were evident in the higher-dose groups (1.175 and 3.290 mg/rabbit), manifesting as characteristic central nervous system disturbances, including an arched posture, muscular rigidity, tremors, vocalizations, and intense struggling (Figure 4C). These findings establish a clear dose–response relationship between the sodium citrate administration and neurological manifestations.

Notably, the observed neurological symptoms were self-limiting within 1–2 h post-administration, mirroring the transient nature of clinical adverse events. The biochemical analysis revealed marked alterations in cerebrospinal fluid (CSF) calcium and magnesium levels at the 3.290 mg/rabbit dosage, suggesting that citrate’s metal-chelating properties may contribute to these neurological disturbances. Further details regarding the detection methods, methodological validation, and complete results are provided in the Supplementary Materials. This hypothesis is corroborated by the existing literature on citrate-mediated ion chelation [40,41].

The histopathological evaluation of administration sites across all dose groups showed no evidence of local tissue damage or structural abnormalities (HE-stained spinal cord sections of New Zealand rabbits were provided in Supplementary Figure S1). The absence of histopathological changes despite functional neurological manifestations implies that citrate’s neurotoxic effects may primarily derive from transient biochemical interactions rather than a permanent tissue injury.

## 4. Discussion

Our experimental data establish that sodium citrate induces concentration-dependent neurotoxicity in intrathecal administration models. We observed a robust correlation between the citrate concentration and neurological deficits in the lumbosacral region, paralleled by cerebrospinal fluid calcium depletion and motor neuron viability loss. These effects demonstrate a causal mechanistic relationship between citrate’s high-affinity calcium chelation (Kd = 2.1 nM at physiological pH) and the neural injury. These findings reveal previously unrecognized molecular mechanisms of neural vulnerability mediated through calcium chelation dynamics [3,42]. Building on these mechanistic insights, future investigations should prioritize elucidating time-dependent calcium dysregulation via single-neuron electrophysiology while concurrently developing compartmental pharmacokinetic models of the CSF citrate clearance to predict neurotoxic accumulation thresholds. This foundational work must be integrated with a quantitative analysis of calcium-chelating equivalents and the accelerated development of alternative buffering systems. For translational relevance, strict concentration ceilings should be established for single-bolus administrations with mandatory post-procedural neurofunctional monitoring. As a critical precaution, these findings warrant the discontinuation of citrate buffers in continuous infusion devices and the implementation of enhanced pharmacovigilance protocols—particularly for vulnerable populations and off-label administration routes—incorporating standardized neurofunctional assessments and CSF ion monitoring during formulation development.

## Figures and Tables

**Table 1:** Dose-dependent behavioral and physiological responses in mice after bilateral intrathecal injections.

Single Dose (μg/g)	Phase	Time Point	Initial (n)	Survival (n)	Tail Twisting	Convulsions	Hindlimb Rigidity	Hindlimb Weakness	Locomotor Abnormality	Recovery Time
Saline	First	Imm.	20	20	−	−	−	−	−	/
		1 h		20	−	−	−	−	−	/
		24 h		20	−	−	−	−	−	/
	Second	Imm.	20	20	−	−	−	−	−	/
		1 h		20	−	−	−	−	−	/
		24 h		20	−	−	−	−	−	/
1.833	First	Imm.	20	20	+	−	+	+	+	30 s
		1 h		20	−	−	−	−	−	/
		24 h		20	−	−	−	−	−	/
	Second	Imm.	20	20	+	−	+	+	+	1 min
		1 h		20	−	−	−	−	−	/
		24 h		20	−	−	−	−	−	/
3.666	First	Imm.	20	20	++	−	++	+	+	3 min
		1 h		20	−	−	−	+	+	/
		24 h		20	−	−	−	+	+	/
	Second	Imm.	20	18	++	−	++	+	+	3 min
		1 h	18	18	−	−	+	+	+	/
		24 h	18	18	−	−	−	+	−	/
7.332	First	Imm.	20	15	++	+	++	+	+	10 min
		1 h	15	14	−	−	−	+	+	/
		24 h	14	14	−	−	−	+	+	/
	Second	Imm.	14	8	++	+	++	+	+	10 min
		1 h	8	8	−	−	+	+	+	/
		24 h	8	8	−	−	−	+	+	/
14.664	First	Imm.	20	0	+++	+++	+++	+++	+++	/

Note: Observation scale: −: Not observed; +: Slight (observed in few mice); ++: Obvious; +++: Severe.

## Data Availability

Data are contained within the article.

## Supplementary Materials

The following supporting information can be downloaded at: https://www.mdpi.com/article/10.3390/pharmaceutics17091112/s1, Figure S1: HE-stained spinal cord sections of New Zealand rabbits; Table S1: Standard Curve and Accuracy deviation range; Table S2: Intra-batch accuracy and precision of calcium; Table S3: Intra-batch accuracy and precision of magnesium; Table S4: Total calcium content in cerebrospinal fluid of New Zealand rabbits; Table S5: Total magnesium content in cerebrospinal fluid of New Zealand rabbits.
