# Supplementary material for: Neurotoxicological Evaluation of Intrathecal Citrate Excipients: Calcium Homeostasis Disruption and Safety Implications in CNS Drug Delivery
Source: Pharmaceutics. 2025 Aug 27;17(9):1112. doi: 10.3390/pharmaceutics17091112 (PMC12473437; doi:10.3390/pharmaceutics17091112)
Supplement: Supplementary file 1 [file pharmaceutics-17-01112-s001.zip › pharmaceutics-3787098-supplementary.pdf]

Methods and materials

1. Animals

New Zealand rabbits (conventional grade; 2–4 months old; 2.1–2.6 kg body weight) of both sexes (50% male, 50% female) were used in this study. The animals, sourced from Qingdao Kangda Aibo Biotechnology Co., Ltd. (China) – an accredited laboratory animal provider (Quality Certification No. 370823240100197621) – were non-transgenic wild-types and serologically confirmed pathogen-free. All rabbits were individually housed in stainless-steel cages supplemented with environmental enrichment (e.g., toys) under standardized conditions (SOP-AC-087.02, Jiangsu Dingtai Pharmaceutical Research Co., Ltd.). Animals received commercial diet (Ke'ao Xieli Feed Co., Ltd.) *ad libitum*. Following a 7-day acclimation period, experimental procedures were initiated. In this investigation, four intrathecal sodium citrate dose groups were established in rabbits: 0.104, 0.292, 1.175, and 3.290 mg/rabbit (n = 6 per group, equal gender distribution). The saline group was used as the control group.

2. Intrathecal injection

One day prior to the experiment, the dorsal lumbar region of each New Zealand White rabbit was shaved using electric clippers. On the experimental day, anesthesia was induced with 5% isoflurane and maintained with 3% isoflurane. Under anesthesia, an intrathecal injection was administered between the L5-L6 vertebrae, where test compounds (0.104, 0.292, 1.175, or 3.290 mg/rabbit) or saline (0.1 mL/rabbit) were slowly infused over 5 minutes before gradual needle withdrawal. For pre-dosing cerebrospinal fluid (CSF) collection, approximately 0.5 mL CSF was obtained via cisterna magna puncture using the same anesthesia protocol. Post-dosing CSF collection employed anesthesia through intramuscular injection of ketamine hydrochloride (30 mg/kg) and xylazine hydrochloride (3 mg/kg). Following final CSF collection, euthanasia was performed via femoral artery exsanguination. Spinal

cord tissue surrounding the injection site and corresponding dorsal root ganglia were harvested, fixed in 10% neutral buffered formalin, then processed through dehydration, paraffin embedding, sectioning, hematoxylin and eosin (H&E) staining, and coverslipping for histological examination.

3. Determination of calcium and magnesium content

3.1 Solution Preparation

Sample diluent was prepared by diluting a mixture of 500 μL 95% HNO_3, 50 μL Triton™ X-100, and 2 mL methanol to 100 mL with ultrapure water. Calibration standards (5–400 ng/mL for Ca/Mg) were generated through serial dilution of primary stock solutions (100 μg/mL), with intermediate standards at 10 μg/mL (Standard I) and 1 μg/mL (Standard II). Quality control (QC) samples were independently prepared at 15, 75, and 300 ng/mL. Intra-batch accuracy and precision were determined by comparing calculated concentrations from daily calibration curves against nominal QC values.

3.2 Sample pretreatment

A 100 μL aliquot of cerebrospinal fluid sample was mixed with 200 μL of 95% nitric acid solution and heated in a 65°C water bath for 2 hours. Following heating, 7,700 μL of sample diluent was accurately added and mixed to yield an 80-fold diluted sample solution. The mixture was then centrifuged at 15,000 rpm for 10 minutes, and the resulting supernatant was transferred for ICP-MS analysis.

Results

1. Method validation

1.1 Standard Curve

Tab1. Standard Curve and Accuracy deviation range

Determined element	Linear range (ng/mL)	Regression equation	Correlation coefficient (r^2)	Accuracy deviation range
Ca	5-400	y=2x+0	0.997	-9.24%~0.00%
Mg	5-400	y=86x+0	0.991	-11.77%~7.11%

1.2 Intra-batch accuracy and precision

With the exception of one calcium MQC sample and one magnesium LQC sample falling outside the acceptable accuracy range (±15%) , all other QC samples met the accuracy criterion (±15%). The number of QC replicates at each concentration level satisfied the requirement of being greater than half

(>1/2). Furthermore, 17 out of all samples analyzed fell within the acceptable accuracy range, exceeding the requirement of two-thirds (>2/3). The coefficients of variation (CV) for low, medium, and high-concentration QC samples all met the criterion of CV ≤ 15%. In summary, the method for calcium and magnesium determination demonstrated good intra-batch accuracy and precision.

Tab 2. Intra-batch accuracy and precision of calcium

QC Sample	Concentration（ng/mL）	Measured Concentration（ng/mL）	Accuracy deviation（%）	RSD（%）	Average（ng/mL）	CV（%）
LQC	15	14.27	-4.89	0.83	14.64	5.65
		14.55	-3.00			
		14.13	-5.83			
		13.98	-6.77			
		14.69	-2.06			
		16.24	8.30			
MQC	75	74.44	-0.74	5.36	79.43	6.75
		84.05	12.07			
		73.60	-1.87			
		87.16	16.21			
		77.55	3.40			
		79.81	6.42			
HQC	300	275.32	-8.23	4.75	270.52	1.75
		270.80	-9.73			
		262.46	-12.51			
		267.83	-10.72			
		274.19	-8.60			
		272.49	-9.17			

Tab 3. Intra-batch accuracy and precision of magnesium

QC Sample	Concentration（ng/mL）	Measured Concentration（ng/mL）	Accuracy deviation（%）	RSD（%）	Average（ng/mL）	CV（%）
LQC	15	14.12	-5.86	0.47	13.46	3.53
		12.74	-15.09			
		13.72	-8.50			
		13.27	-11.51			
		13.63	-9.11			
		13.29	-11.43			
MQC	75	70.00	-6.67	0.90	69.65	1.29

		70.65	-5.80			
		70.44	-6.08			
		68.75	-8.33			
		69.63	-7.15			
		68.43	-8.76			
HQC	300	278.29	-7.24	2.34	276.26	0.85
		277.98	-7.34			
		276.89	-7.70			
		275.47	-8.18			
		276.98	-7.67			
		271.94	-9.35			

1.3 Total Ca and Mg Quantification in Rabbit CSF by ICP-MS

Tab 4. Total calcium content in cerebrospinal fluid of New Zealand rabbits

Sex	Dose，mg/rabbit	Measured Concentration，mg/L				Rate of change (D1-2h vs Baseline),%
		Rabbit ID	D-1 (Baseline)	D1-0h/1h	D1-2h	
Male	Saline	1001	20.09	-	18.90	-5.92
		1002	21.71	-	40.98	+88.76
		1003	17.35	-	22.17	+27.78
	0.104	1101	18.08	-	32.89	+81.91
		1102	18.08	-	18.73	+3.60
		1103	18.08	-	18.28	+1.11
	0.292	1201	19.30	-	19.25	-0.26
		1202	17.39	-	18.35	+5.52
		1203	18.08	-	19.14	+5.86
	1.175	1301	17.20	-	18.04	+4.88
		1302	-	19.04	22.22	NA

Sex	Dose，mg/rabbit	Measured Concentration，mg/L				Rate of change (D1-2h vs Baseline),%
		Rabbit ID	D-1 (Baseline)	D1-0h/1h	D1-2h	
		1303	18.07	-	17.63	-2.43
	3.290	1502	18.12		39.30	+116.89
		1503	17.43		31.43	+80.32
Female	Saline	2001	18.82	-	16.99	-9.72
		2002	19.46	-	19.13	-1.70
		2003	19.60	-	19.40	-1.02
	0.104	2101	19.13	-	20.43	+6.80
		2102	18.43	-	20.81	+12.91
		2103	20.42	-	19.70	-3.53
	0.292	2201	19.24	-	19.74	+2.60
		2202	18.04	-	18.95	+5.04
		2203	20.22	-	18.64	-7.81
	1.175	2301	17.45	-	17.60	+0.86

Sex	Dose，mg/rabbit	Measured Concentration，mg/L				Rate of change (D1-2h vs Baseline),%
		Rabbit ID	D-1 (Baseline)	D1-0h/1h	D1-2h	
		2302	19.88	-	18.51	-6.89
		2302	18.11	-	17.96	-0.83
	3.290	2501	18.24	-	-	+4.06
		2502	18.43	-	26.69	+44.82
		2503	17.69	24.24		+37.03

Note: The symbol "-" indicates time points where no sampling occurred.

Tab 5. Total magnesium content in cerebrospinal fluid of New Zealand rabbits

Sex	Dose，mg/rabbit	Measured Concentration，mg/L				Rate of change (D1-2h vs Baseline),%
		Rabbit ID	D-1 (Baseline)	D1-0h/1h	D1-2h	
**Male**	Saline	1001	18.94	-	20.43	+7.9
		1002	19.55	-	20.37	+4.2
		1003	18.97	-	19.36	+2.1

Sex	Dose，mg/rabbit	Measured Concentration，mg/L				Rate of change (D1-2h vs Baseline),%
		Rabbit ID	D-1 (Baseline)	D1-0h/1h	D1-2h	
	0.104	1101	19.67	-	23.64	+20.2
		1102	20.15	-	29.35	+45.7
		1103	20.50	-	20.43	-0.3
	0.292	1201	19.59	-	19.62	+0.2
		1202	19.15	-	29.64	+54.8
		1203	20.73	-	21.71	+4.7
	1.175	1301	20.80	-	21.98	+5.7
		1302	-	-	29.43	NA
		1303	20.67	-	20.61	-0.3
	3.290	1401	20.99	-	25.31	+20.6
		1402	20.10	-	24.49	+21.8
		1403	19.99	-	26.43	+32.2
**Female**	Saline	2001	20.39	-	18.40	-9.8

Sex	Dose，mg/rabbit	Measured Concentration，mg/L				Rate of change (D1-2h vs Baseline),%
		Rabbit ID	D-1 (Baseline)	D1-0h/1h	D1-2h	
		2002	21.18	-	20.74	-2.1
		2003	21.19	-	21.91	+3.4
	0.104	2101	19.98	-	19.86	-0.6
		2102	21.48	-	22.01	+2.5
		2103	20.72	-	21.29	+2.8
	0.292	2201	20.86	-	20.97	+0.5
		2202	21.57	-	20.32	-5.8
		2203	20.70	-	20.77	+0.3
	1.175	2301	21.15	-	21.65	+2.4
		2302	20.68	-	22.38	+8.2
		2302	20.69	-	21.16	+2.3
	3.290	2501	20.41	-	21.58	+5.7
		2502	20.22	-	21.31	+5.4

Sex	Dose，mg/rabbit	Measured Concentration，mg/L				Rate of change (D1-2h vs Baseline),%
		Rabbit ID	D-1 (Baseline)	D1-0h/1h	D1-2h	
		2503	19.09	29.50	-	NA

Note: The symbol "-" indicates time points where no sampling occurred.

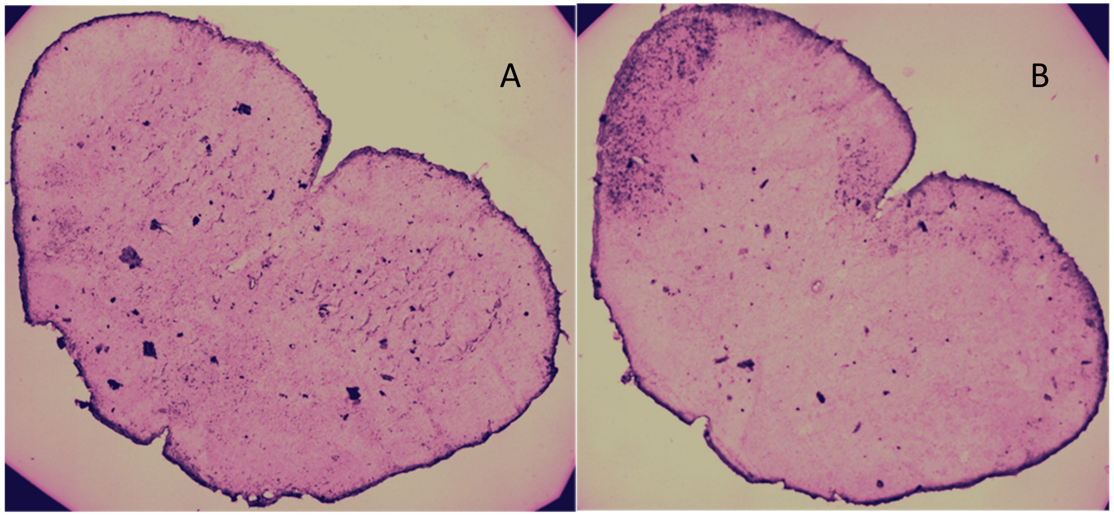

Figure S1 HE-stained spinal cord sections of New Zealand rabbits. A.SC group; B. Saline group
